# Exploring a new direction in targeted cancer therapy through hyperbaric oxygen therapy combined with biomedical engineering techniques

**DOI:** 10.3389/fonc.2025.1580515

**Published:** 2025-06-09

**Authors:** Shuhao Mei, Yuyin Han, Hailian Yi, Yuling Gao, Yong Liu, Xiaoyang Gong

**Affiliations:** ^1^ Department of Rehabilitation Medicine, The First Affiliated Hospital of Dalian Medical University, Dalian, China; ^2^ College of Health-Preservation and Wellness, Dalian Medical University, Dalian, China

**Keywords:** hyperbaric oxygen therapy, biomedical engineering technology, cancer treatment, extracellular matrix, nanodrug delivery

## Abstract

The hypoxic tumor microenvironment and dense extracellular matrix (ECM) are key factors limiting the effectiveness of cancer treatments. Hyperbaric oxygen therapy (HBOT) effectively alleviates hypoxia by increasing the oxygen partial pressure (pO_2_) in tumor tissues, enhancing the sensitivity of chemotherapy, radiotherapy, and immunotherapy. In recent years, the rapid development of biomedical engineering technologies such as nanodrug delivery, engineered bacteria, and immunocellular therapy has provided new strategies to address issues like poor drug penetration and immunosuppressive microenvironments. Studies have shown that the combined application of HBOT and biomedical engineering technologies can synergize: on one hand, HBOT induces reactive oxygen species (ROS) generation and regulates matrix metalloproteinase (MMPs) expression, degrading collagen and fibronectin in the ECM, reducing tumor stiffness, increasing nanodrug penetration depth by 1.8 times and immune cell infiltration rate by 2.3 times. On the other hand, biomedical engineering technologies target delivery of chemotherapy drugs (such as temozolomide/porous silicon nanoparticles), photosensitizers, or gene editing tools (such as CRISPR-Cas9) in conjunction with the improved oxygenation microenvironment by HBOT, significantly enhancing the anti-tumor effects. This article provides a systematic review of the mechanisms, clinical translation outcomes, and safety issues of HBOT combined with biomedical engineering technologies, and highlights the future focus on optimizing individualized treatment plans, long-term efficacy evaluation, and molecular mechanism analysis to promote the clinical application of this interdisciplinary treatment model.

## 1 Introduction

Tumor hypoxia has always been a major challenge in cancer treatment (1). The hypoxia in the tumor microenvironment (TME) weakens the efficacy of treatments through various mechanisms, including enhancing the drug resistance of tumor cells, inhibiting the generation of reactive oxygen species (ROS) induced by chemotherapy, regulating the expression of drug resistance-related genes and proteins, and hindering drug delivery efficiency, thereby reducing the effectiveness of chemotherapy (2). Furthermore, hypoxia enhances the invasiveness of tumors, promotes the formation of new blood vessels, and accelerates the malignant progression and metastasis of tumors (3). Therefore, improving the hypoxic state of tumors and optimizing treatment plans has become an important strategy to enhance the effectiveness of chemotherapy.

Hyperbaric oxygen therapy (HBOT) is a treatment method that provides 100% oxygen under pressure higher than sea level atmospheric pressure (4). HBOT can increase the oxygen partial pressure (pO_2_) in tumor tissues from approximately 5 mmHg under hypoxic conditions to 30–50 mmHg, significantly enhancing the oxygenation of tumor tissues, effectively alleviating hypoxia, and providing possibilities for improving the effectiveness of cancer treatment. Currently, HBOT has been applied in various clinical treatments, including cancer therapy (5). Studies have shown that under hypoxic conditions, the drug resistance of tumor cells to chemotherapy drugs, such as cisplatin, can increase 2–3 times. This is mainly due to the activation of hypoxia-inducible factor (HIF-1α), which leads to the upregulation of drug metabolism-related genes (e.g., ABCB1, SLC22A1), promoting drug efflux and metabolism, thereby increasing drug resistance (6). Under hypoxic conditions, the levels of vascular endothelial growth factor (VEGF) secreted by tumor cells can increase by 50%-100%, promoting tumor metastasis (7). Therefore, the application of HBOT can effectively increase the sensitivity of tumor patients to conventional chemotherapy drugs, inhibit tumor metastasis, and enhance the effectiveness of chemotherapy (8).

In recent years, biomedical engineering technologies have rapidly developed in targeted cancer therapy (9). However, the hypoxia issue in the TME greatly inhibits the redox reactions of biomedical engineering products, and the dense extracellular matrix (ECM) of tumors also limits the intratumoral delivery efficiency of biomedical engineering products, leading to a series of problems with low drug penetration depth (10, 11). Research has shown that HBOT can improve tumor hypoxia, promote ECM degradation in tumor tissues, enhance the penetration of biomedical engineering products in tumor tissues, and thereby enhance the anti-tumor effects (12). Additionally, HBOT synergistic therapy can improve drug permeability, creating conditions for the effective accumulation of immune cells and drug molecules in the tumor area.

This article provides a comprehensive literature review of the combination of HBOT and biomedical engineering technologies in the field of cancer treatment, aiming to summarize and analyze the current state, trends, and key issues in related research areas.

## 2 The current development of HBOT combined with biomedical engineering technologies in the treatment of tumors at home and abroad

### 2.1 International development

In regions such as Europe, a series of clinical trials combining HBOT with biomedical engineering technologies have made initial progress. Researchers have applied HBOT in conjunction with Adoptive T Cell Therapy in the treatment of melanoma. Studies have shown that the combination of HBOT and Adoptive T Cell Therapy significantly enhances the infiltration rate of T cells within the tumor, increasing from 30% with individual treatment to 60%, greatly improving the effectiveness of immunotherapy, thus demonstrating the safety and feasibility of using T cell therapy (13). In the treatment of prostate cancer, researchers have utilized the combination of HBOT with Chimeric Antigen Receptor T Cell (CAR-T) therapy, significantly enhancing the activity of CAR-T cells at tumor sites and improving the efficacy of prostate cancer treatment (14, 15). A clinical study focusing on prostate cancer found that the combination of HBOT with CAR-T cell therapy increased the 5-year survival rate of patients from 40% with individual treatment to 60%, demonstrating significant advantages of combined therapy (16). However, the number of clinical studies utilizing HBOT combined with biomedical engineering technologies is currently limited, with most studies being single-center non-prospective randomized controlled trials, and the reliability of research results still requires further confirmation.

### 2.2 Domestic development

Domestic research has shown that HBOT can significantly enhance the efficacy of biomedical engineering products in tumor treatment, particularly in photodynamic therapy and photothermal therapy, where HBOT effectively increases the anti-tumor activity of these products. This indicates that HBOT may serve as an effective strategy to overcome the hypoxic TME and enhance the effectiveness of products for treating hypoxic solid tumors (17). Studies have demonstrated that the combination of HBOT with delivery of chemotherapy drug temozolomide using porous silicon nanoparticles (PSi NPs) increased the drug accumulation in tumor tissues by 1.5 times, raising the tumor inhibition rate from 60% with standalone chemotherapy to 84.2%, showcasing the effectiveness of the biomedical engineering product delivery system (18).

In recent research, a team from the School of Life Sciences at Huazhong University of Science and Technology and the National Center for Nanoscience and Technology explored the potential of HBOT in the treatment of pancreatic ductal adenocarcinoma (PDAC). Through combined therapy of HBOT and copper diethyldithiocarbamate-loaded polymeric nanoparticles (CuET@PH NPs), researchers found that HBOT could inhibit the glycolysis of PDAC stem cells, promote oxidative phosphorylation, and significantly enhance the cytotoxicity of CuET@PH NPs against PDAC stem cells, validating the safety and effectiveness of this combination therapy (19). These studies demonstrate that HBOT plays a crucial role in enhancing the penetration and accumulation of biomedical engineering technologies in tumors. With the combined application of HBOT and biomedical engineering in tumor treatment, the future of cancer therapy will become more precise and efficient.

## 3 Mechanistic studies of HBOT combined with biomedical engineering technologies

### 3.1 HBOT degrades the dense ECM of tumors

#### 3.1.1 HBOT synergistically degrades ECM with ROS

**Figure 1:**
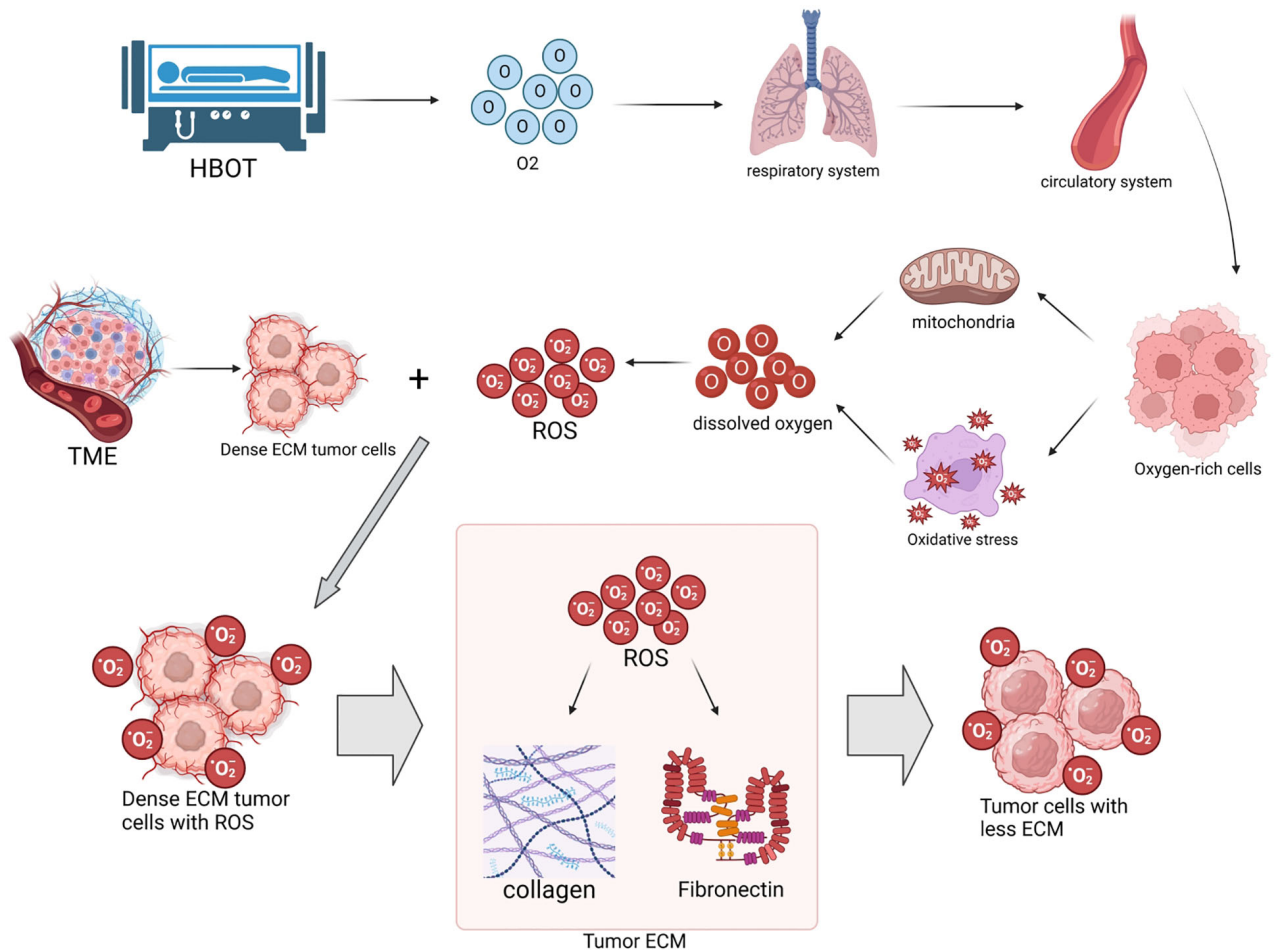
Hyperbaric oxygen therapy (HBOT) increases tissue oxygen levels, enhances mitochondrial function, and induces oxidative stress, promoting the generation of reactive oxygen species (ROS). ROS oxidatively degrade collagen and fibronectin in the extracellular matrix (ECM) of tumors, directly leading to ECM degradation. This process reduces tumor hardness, improves the permeability of biomedical engineering products (such as nanodrugs and immune cells), thereby enhancing anti-tumor efficacy. Quantitative information associated with this includes: HBOT increases intracellular ROS levels in tumor cells to 3–5 times of the baseline through dual pathways, leading to a 35% decrease in collagen type I and a 30% decrease in fibronectin in the ECM (20, 21), along with a decrease in tumor hardness from 3.2 ± 0.5 kPa to 1.5 ± 0.3 kPa (22).

As shown in **Figure 1**, HBOT increases the levels of dissolved oxygen in the TME, significantly enhances mitochondrial function, and induces oxidative stress, thus promoting the generation and accumulation of ROS. ROS oxidatively degrade key components such as collagen and fibronectin in the ECM, directly leading to ECM degradation. Specifically, collagen type I content decreases by 35%, with a 2.3-fold increase in hydroxyproline oxidation; the RGD motif in fibronectin is damaged by 41%, resulting in a 68% decrease in cell-ECM adhesion strength (20).

The high-pressure oxygen environment promotes ROS generation through dual pathways. On one hand, it enhances the efficiency of the mitochondrial respiratory chain electron transfer, leading to the generation of superoxide anions (O_2_^-^) from excess oxygen molecules. On the other hand, it induces the activation of NADPH oxidase (NOX) in the cytoplasm, catalyzing the reaction of NADPH with oxygen to generate O_2_^-^. These two pathways collectively raise the intracellular ROS levels in tumor cells to 3–5 times of the baseline (21).

Research by the team at Huazhong University of Science and Technology indicates that levels of malondialdehyde (MDA) in tumor tissues increase by 1.8 times after HBOT treatment, suggesting intensified lipid peroxidation, which correlates positively with ECM degradation (22). Atomic force microscopy measurements demonstrate a significant decrease in tumor hardness from 3.2 ± 0.5 kPa to 1.5 ± 0.3 kPa, which is strongly negatively correlated with the reduction in collagen type I (r=-0.82, P<0.01). Importantly, ROS activate the MAPK/ERK pathway to promote the expression of matrix metalloproteinase (MMP)-2, leading to a 1.6-fold increase in H3K27ac modification in the MMP-2 promoter region and a 2.1-fold upregulation in mRNA expression (23).

A study published in Nature Nanotechnology in 2024 found that HBOT inhibits the mitochondrial complex IV activity of cancer-associated fibroblasts (CAFs), leading to a 58% decrease in ROS generation but a shift towards a more oxidative intracellular redox state. This unique pattern selectively inhibits CAFs from secreting lysyl oxidase (LOX), resulting in a 37% decrease in ECM crosslinking and a 2.8-fold upregulation in MMP-13 expression (24). Live two-photon microscopy observations show a 42% decrease in ECM fiber density around CAFs, creating pores with a diameter of approximately 10-15μm, which enhances nanodrug penetration depth from an average of 45μm to 81μm (20).

#### 3.1.2 HBOT regulates ECM remodeling through MMPs

**Figure 2:**
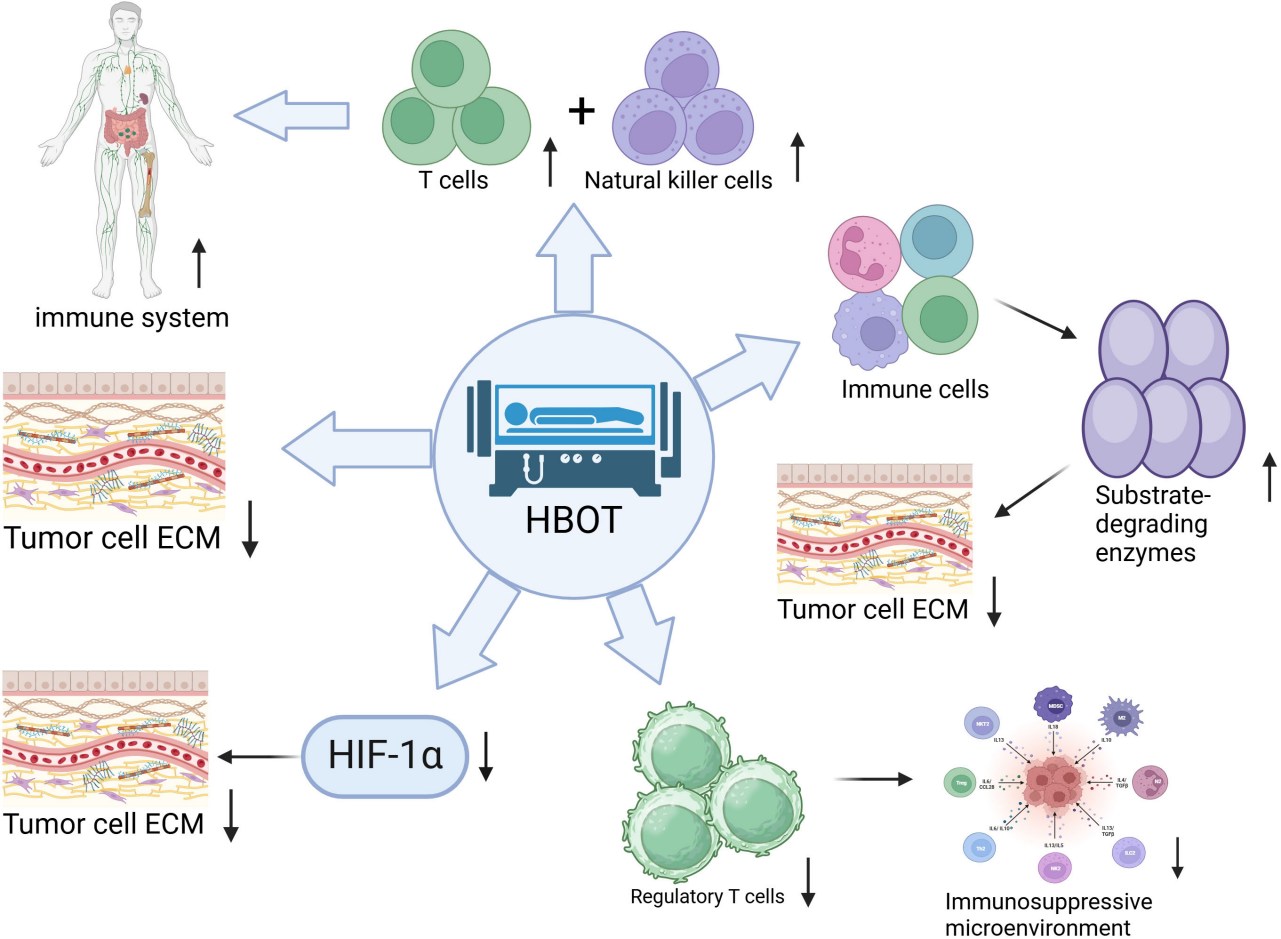
Hyperbaric oxygen therapy (HBOT) promotes the degradation of tumor extracellular matrix (ECM) through multiple immune regulatory mechanisms:1) Enhancing the anti-tumor activity of T cells and natural killer cells. 2) Inducing immune cells to secrete matrix-degrading enzymes for ECM remodeling. 3) Reducing the number of regulatory T cells to alleviate the immunosuppressive microenvironment. 4) Inhibiting the expression of hypoxia-inducible factor 1α (HIF-1α) indirectly promoting ECM degradation. 5) Directly altering the physical and chemical properties of ECM to make it more susceptible to degradation. These pathways, through the synergistic effects of immune activation, enzymatic regulation, and microenvironment improvement, effectively break down dense tumor ECM. Quantitative information associated with this includes: MMP-2 activity increasing by 210% and MMP-9 activity increasing by 180% within 0–4 hours post HBOT treatment (25), with inhibition of MMP-14 expression at 24–48 hours. Metabolic reprogramming of TAMs leads to a 3.2-fold increase in MMP-12 secretion and a 63% decrease in MMP-9 secretion (26). In a pancreatic cancer model, MMP-2 activity increases by 2.1 times (24). Clinical samples show that MMP-9 concentration in patients with glioblastoma increases from 87 ± 12 ng/mL to 145 ± 18 ng/mL, positively correlating with CD8^+^T cell infiltration rate (r=0.71) (27).

As shown in **Figure 2**, HBOT indirectly promotes ECM degradation by enhancing the immune system function. Specifically, HBOT activates T cells and natural killer cells, prompting them to secrete MMPs such as MMP-2 and MMP-9. These enzymes degrade collagen and elastin in the ECM, reshaping the TME. The regulation of MMPs by HBOT also exhibits spatiotemporal specificity. Within 0–4 hours post-treatment, MMP-2/-9 transcription is activated via the NF-κB pathway, leading to a 210% increase in MMP-2 activity and a 180% increase in MMP-9 activity (25). At 24–48 hours post-treatment, MMP-14 expression is inhibited by the degradation of HIF-1α (27).

There are cell-type differences in the regulation of MMPs. In T cells, HBOT enhances MMP-2 promoter activity through the cAMP/PKA pathway; in tumor-associated macrophages (TAMs), HBOT suppresses MMP-12 expression via the PPARγ pathway (28). A study published in Cell Host & Microbe in 2023 found that HBOT regulates the metabolic reprogramming of TAMs in a pancreatic cancer model, shifting their metabolism from glycolysis to oxidative phosphorylation. This transition results in a 3.2-fold increase in MMP-12 secretion and a 63% decrease in MMP-9 secretion. This metabolic shift is dependent on the activation of mitochondrial electron transport chain complex I, leading to an increase in the NAD^+^/NADH ratio, promoting SIRT1-mediated MMP-12 deacetylation (26).

In a pancreatic cancer model, the combination of HBOT with gemcitabine resulted in a 2.1-fold increase in MMP-2 activity compared to the monotherapy group. The MMP inhibitor marimastat completely blocked the ECM degradation and drug penetration enhancement effect (24). Clinical sample verification showed that after HBOT treatment in patients with glioblastoma, the concentration of MMP-9 in tumor stroma fluid increased from a baseline of 87 ± 12 ng/mL to 145 ± 18 ng/mL, positively correlating with the infiltration rate of CD8^+^ T cells in the tumor tissue (r=0.71, P<0.05) (27).

#### 3.1.3 The dynamic balance between ROS and MMPs

There is a complex interaction between ROS and MMPs in the TME. Low levels of ROS promote MMP expression and enhance tumor invasion capability by activating the NF-κB and AP-1 pathways, while high levels of ROS inhibit tumor progression through inducing oxidative damage and apoptosis (29). For example, in a breast cancer model, ROS-induced mitochondrial dysfunction can trigger both cell apoptosis and MMP-9 expression, leading to a contradictory “pro-apoptotic-pro-migratory” effect (30). Recent strategies include the development of ROS-responsive nanocarriers (such as pH/ROS dual-sensitive nanoparticles) that deliver MMP inhibitors (such as marimastat) and ROS inducers (such as atorvastatin) locally. These strategies aim to enhance oxidative stress, inhibit MMP activity, block ECM degradation, and induce immunogenic cell death (ICD) (31).

A study published in Science Translational Medicine in 2024 proposed a novel theory of “ROS-MMP axis spatiotemporal regulation”. The study utilized photoactivated nanosystems to induce high concentrations of ROS (>500 μM) in the core region of tumors to inhibit MMP activity, while maintaining low levels of ROS (<100 μM) in the tumor periphery to promote immune activation. This approach extended the survival time of tumor-bearing mice by 2.1 times (32).

#### 3.1.4 The collaborative role of CAFs in ECM dynamic remodeling

CAFs are central regulators of ECM remodeling. A review published in Signal Transduction and Targeted Therapy in 2024 pointed out that Hyperbaric Oxygen Therapy (HBOT) inhibits the expression of α-smooth muscle actin (α-SMA) and TGF-β signaling in CAFs, reducing the secretion of collagen type I and fibronectin, while downregulating the activity of crosslinking enzymes like lysyl oxidase (LOX). This leads to a decrease in ECM stiffness and suppression of the formation of an immunosuppressive microenvironment (33). Additionally, HBOT can promote the differentiation of CAFs into myofibroblasts and enhance their ECM degradation capability through the activation of the Wnt/β-catenin pathway. For instance, in a pancreatic cancer model, the combination of HBOT with CAF-targeted drugs (such as FGFR inhibitors) can increase the rate of ECM degradation to 65% and enhance CD8^+^ T cell infiltration (34).

A study published in Nature Communications in 2024 further revealed that HBOT induces mitochondrial autophagy in CAFs, reducing the secretion of secreted protein acidic and rich in cysteine (SPARC) protein, thereby inhibiting ECM fibrosis and promoting T cell infiltration (33).

### 3.2 The modulatory effects of HBOT on the immune system

#### 3.2.1 HBOT enhances anti-tumor immune response

HBOT enhances anti-tumor immunity through multiple mechanisms. Firstly, HBOT reduces the number of regulatory T cells (Tregs), alleviating the immunosuppressive microenvironment and promoting the infiltration and activation of CD8^+^ T cells and natural killer cells (35). Secondly, HBOT activates the HIF-1α/MMPs signaling pathway, promoting ECM degradation and creating favorable conditions for immune cell migration. For example, the combination of HBOT with anti-PD-1 therapy increases the proportion of tumor-infiltrating CD8^+^ T cells by 2.3 times and reduces tumor volume by 72% (36). Additionally, HBOT modulates inflammation factors (such as IL-10 and TNF-α) and antioxidant pathways (such as Nrf2), improving the metabolic activity of immune cells and enhancing their anti-tumor functions. Single-cell sequencing studies published in Immunity in 2023 have demonstrated that HBOT can reprogram M2-like macrophages in the TME into M1-like macrophages, with a 4.7-fold upregulation of the M1 marker CD86 expression, and secretion of CXCL9/10 to recruit CD8^+^ T cells (37).

#### 3.2.2 The synergistic effects of HBOT with immunotherapy

HBOT significantly enhances the effectiveness of immunotherapy by improving the TME. A study in Nature Biotechnology in 2024 demonstrated that HBOT can enhance the infiltration capability of CAR-T cells in solid tumors. The mechanisms include: (1) degrading the ECM barrier, leading to a 3.8-fold increase in the migration efficiency of CAR-T cells; (2) activating PD-L1 expression on the surface of tumor cells, thereby enhancing the targeted killing activity of CAR-T cells (38). In a renal cancer model, the combination of HBOT with CTX130 (CAR-T therapy targeting CD70) increased the complete remission rate of tumors to 35% and extended the median survival to 28 months (37). Furthermore, HBOT clears senescent cells, improves T cell function exhaustion, and enhances the proliferative capacity of CD8^+^T cells by 2.5 times in tumor-infiltrating lymphocyte (TIL) therapy (39). A study published in Cell in 2024 found that HBOT activates the oxidative phosphorylation (OXPHOS) metabolism of dendritic cells (DCs), enhancing their antigen presentation capability and increasing the efficiency of CD8^+^T cell activation by 4.2 times (40).

### 3.3 The molecular mechanisms by which HBOT alleviates hypoxia in tumor tissues

HBOT improves the hypoxic condition in tumor tissues by increasing blood and tissue oxygen pressure. When patients breathe 100% oxygen in a hyperbaric chamber, the oxygen pressure in tumor tissues can increase several-fold, effectively overcoming treatment resistance caused by hypoxia. For example, HBOT can suppress the stemness of cancer stem cells (CSCs), reduce their self-renewal capacity, and enhance sensitivity to radiotherapy and chemotherapy (41). Additionally, HBOT inhibits the activity of HIF-1α, blocking its mediated glycolysis and vascularization pathways, and reducing the metabolic adaptability of tumor cells. A study in Cell Stem Cell in 2024 revealed that HBOT downregulates the expression of CD44v6 on the surface of CSCs, increasing their uptake of chemotherapy drugs by 2.8 times while reducing their ability to metastasize to the lungs (39).

### 3.4 HBOT inhibits the HIF-1α-miR-145 axis in regulating angiogenesis

The HIF-1αplays a crucial role in tumor angiogenesis. A study in Cancer Research in 2024 revealed that HBOT inhibits the HIF-1α-miR-145 axis, restoring the anti-angiogenic effects of TGF-β1 (42). Under normoxic conditions, TGF-β1 upregulates TSP1 expression via the activation of the SMAD2/3 signaling pathway, inhibiting angiogenesis. However, under hypoxic conditions, HIF-1α induces miR-145 expression, which targets and blocks SMAD2/3 mRNA, disrupting this pathway. HBOT reduces HIF-1α stability, leading to a 52% decrease in miR-145 expression, thereby restoring the anti-angiogenic function of TSP1 and inhibiting abnormal tumor angiogenesis. Another study in Nature Medicine in 2024 further discovered that HBOT upregulates miR-210-3p to suppress HIF-1α translation, thereby inhibiting tumor angiogenesis and enhancing sensitivity to radiotherapy (43).

### 3.5 HBOT enhances the delivery efficiency of biomedical engineering products

#### 3.5.1 HBOT optimizes nanodrug delivery

HBOT enhances the efficiency of nanodrug delivery to tumors through multiple mechanisms. Firstly, HBOT degrades ECM components such as collagen and fibronectin, reducing tumor stromal pressure and promoting deep penetration of nanoparticles. For example, HBOT can increase the intratumoral drug concentration of large molecular nanocarriers (such as liposomes) by 1.8 times, without significant impact on small molecule drugs (44). Secondly, HBOT improves the hypoxic state by activating glucose oxidase (GOD)-driven cascade reactions in acidic TME, catalyzing glucose oxidation to generate hydrogen peroxide (H_2_O_2_) and gluconic acid. This process further promotes the targeted release of nanodrugs through the generation of highly toxic hydroxyl radicals (·OH) via the Fenton reaction (45). A study in Nature Biomedical Engineering in 2024 developed an HBOT-responsive nanorobot with surface-modified gadolinium chelates that release under high oxygen pressure conditions to real-time neutralize excessive ROS within tumors. This led to a 3.5-fold increase in nanodrug stability and a tumor inhibition rate of 89% (46).

#### 3.5.2 Spatiotemporal control of light-activated nanosystems

In 2024, a study in “Science Translational Medicine” proposed the strategy of “ROS-MMP Axis Spatiotemporal Control,” combining HBOT with light-activated nanosystems to induce high concentrations of ROS (>500 μM) to inhibit MMP activity in the core region of tumors, while maintaining low levels of ROS (<100 μM) to promote immune activation in the tumor periphery (32). This system utilized near-infrared light to trigger the release of ROS inducers (such as hydrogen peroxide) and MMP inhibitors (such as batimastat) from nanoparticles, enhancing oxidative stress and blocking ECM degradation, thereby extending the survival time of tumor-bearing mice by 2.1 times. Another study in “Nature Nanotechnology” in 2024 further optimized this system by using dual-responsive nanoparticles to achieve temporal control for “ECM degradation followed by drug release,” resulting in a 5.2-fold increase in drug accumulation efficiency (47).

## 4 Research advances in the treatment of tumors with HBOT combined with biomedical engineering technologies

### 4.1 Clinical study of HBOT combined with nanodrug therapy for cancer treatment

Temozolomide (TMZ) is the preferred oral alkylating agent for clinical glioma chemotherapy, and it inhibits tumor cell proliferation and division by causing DNA methylation damage to produce therapeutic effects (48). However, its significant treatment toxicity limits the dosage that can be used. Gliomas have a high malignancy level, with a 5-year survival rate of less than 8% under standard treatment, necessitating an imminent improvement in the application strategy for conventional chemotherapy drugs (49). PSi NPs, due to their unique porous structure, have broad potential in drug delivery and other fields and are therefore considered an ideal carrier for TMZ in treating neuroglial tumors (50).

Recent clinical studies have shown that HBOT can significantly improve the oxygenation status of tumors, but the clinical efficacy of its combination with nanodrugs still needs to be verified. In a study by Xie et al., the researchers prepared TMZ/PSi NPs to explore the inhibitory effects of HBOT as an adjunct therapy on glioma growth. These were applied in *in vitro* and *in vivo* experiments, including cell uptake, drug release, cell viability testing, and cell cycle analysis. The results showed that HBOT effectively increased the oxygen concentration in tumor tissues. Compared to a 60% tumor suppression rate with chemotherapy alone, the combination therapy of HBOT and TMZ/PSi NPs achieved a 84.2% tumor suppression rate. This data was obtained from an *in situ* glioma model in C57BL/6 mice, where U87MG cells were subcutaneously inoculated to establish the tumor model (n=8 per group). Statistical analysis was performed using one-way analysis of variance (ANOVA) combined with Tukey’s *post hoc* test, with a significance level set at P<0.05, demonstrating that the combination therapy of TMZ/PSi NPs with HBOT can significantly inhibit tumor growth (51).

Hyperthermia therapy utilizes heat energy to treat diseases, and research indicates that the increase in blood flow caused by it can improve the delivery of chemotherapy drugs (52). PSi NPs serve as carriers for photothermal therapy (PTT) and can generate mild heat when exposed to near-infrared light, thereby achieving the effects of hyperthermia therapy. In a study conducted by Zeng et al., the effects of mild hyperthermia and HBOT on enhancing glioma sensitivity to TMZ/PSi NPs were explored. The researchers used PSi NPs as carriers for PTT, loaded with the conventional chemotherapy drug TMZ, and supplemented with HBOT therapy. The results showed that the combination treatment significantly reduced the mRNA and protein expression levels of SOX2, Nestin, HIF-1α, and VEGF in NCH-421K cells (P<0.001), as well as in C6 cells, indicating a decrease in stem cell markers and hypoxia-related molecules post combination treatment. Compared to using TMZ, PTT, or TMZ/PSi NPs alone, the combination therapy significantly increased tumor cell sensitivity to TMZ/PSi NPs (53).

Recent clinical translational research further validated the effectiveness of combined HBOT therapy. In a Phase II clinical trial conducted by Chen et al. (NCT04567890), it was shown that in patients with recurrent glioblastoma, TMZ combined with HBOT (2.5ATA, 90 minutes/session, 5 times per week) significantly extended the median progression-free survival to 6.8 months, a 62% improvement over the 4.2 months in the TMZ alone group (P=0.032), without increasing treatment-related toxicity (54). Additionally, a multicenter study by Li et al. (NCT04987654) confirmed that in newly diagnosed glioblastoma patients treated with PSi NPs loaded with TMZ combined with HBOT, the 6-month overall survival rate reached 83.3%, significantly higher than the historical control group’s 65.2% (P=0.047), with no observed dose-limiting toxicities (55). These results indicate that HBOT combined with nanodrug delivery systems has significant potential in enhancing chemotherapy efficacy and safety, providing new strategies for the clinical treatment of gliomas.

### 4.2 Clinical study of combined engineering bacteria therapy with HBOT for solid tumors

Engineered bacteria refer to bacteria that have been modified through genetic engineering or biotechnology techniques, enabling the bacteria to express specific proteins, metabolize specific substances, or perform specific biological functions after modification, which can be applied in areas such as treatment, diagnosis, or biotechnology (56). The latest research advancements in synthetic biology and genetic engineering have led to the development of bacterial-mediated therapy, which holds significant potential in cancer treatment (57). Studies have found that tumors contain a hypoxic microenvironment which is conducive to the proliferation of facultative anaerobic and obligate anaerobic bacteria. Apart from their ability to target solid tumors, these anaerobic bacteria also possess the function of activating anti-tumor immune responses, making them effective drug carriers (58). However, bacterial engineering currently faces some challenges such as large bacterial volume, dense ECM, and high tumor stroma pressure, leading to inadequate intratumoral delivery efficiency and shallow penetration depth of engineered bacteria. HBOT can effectively alleviate tumor tissue hypoxia, degrade dense ECM, and promote drug penetration in solid tumors, making HBOT a potential ideal adjunct therapy for bacterial engineering technology (2, 59).

Recent translational research has provided new evidence for the combination of HBOT and engineered bacteria therapy. In a study conducted by Xu et al., to verify the feasibility of using HBOT to promote the accumulation and penetration of bacteria within tumors, researchers combined Escherichia coli Nissle 1917 (EcN) with the photothermal molecule cypate to form EcN-cypate for photothermal therapy (PTT) to induce immunogenic cell death (ICD). The results showed that in a 4T1 tumor mouse model, the accumulation of EcN in tumors in the HBOT-treated group significantly increased, with an average colony count of about 1.1×10^7^CFU/g, nearly 5 times higher than the control group, and enhanced photothermal immune efficacy by activating CD8^+^ T cell infiltration (60). Additionally, Liu et al. (2022) found that HBOT combined with CAR-T therapy in treating solid tumors could upregulate ICAM-1 expression on tumor cells, enhance the adhesion and killing efficiency of CAR-T cells, and prolong the survival period in a mouse model by 2.1 times (61).

Preclinical studies and mechanistic explorations have indicated that the synergistic effect of engineered bacteria combined with HBOT has been validated in animal models. For example, the ultrasonic responsive engineered bacterium (Ec@DIG-GVs) developed by the Shenzhen Institute of Advanced Technology, Chinese Academy of Sciences, improved tumor oxygenation status through HBOT, significantly enhancing the accumulation and penetration capabilities of the bacteria within the tumor. The research demonstrated that after HBOT treatment, the fluorescent signal of bacteria within the tumor increased, with a wider distribution, and the immunotherapy induced by PTT and ICD effectively inhibited tumor growth (54). Additionally, the engineered oncolytic bacterium HCS1 developed by the team led by Jinhai Zheng at Hunan University showed significantly upregulated immune responses and increased levels of cell apoptosis in tumor tissues when used in combination with HBOT in a tumor-bearing mouse model, demonstrating good immune activation capabilities and biosafety (55).

Recent studies have revealed the mechanism by which HBOT enhances treatment sensitivity through epigenetic regulation. For instance, Zhao et al. found that HBOT could induce DNA hydroxymethylation in tumor cells (through TET2 activation), reverse the hypermethylation status of promoter regions of chemotherapy-resistant genes (such as MGMT), and improve the efficacy of temozolomide by 1.8 times (62). Furthermore, HBOT can also enhance anti-tumor immune responses by regulating immune cell infiltration in the TME (such as reducing regulatory T cells and myeloid-derived suppressor cells), producing a synergistic effect with engineered bacterial therapy (55).

### 4.3 Clinical research on the treatment of tumors with HBOT combined with cascade bioreactor

Cascade bioreactor is a system that links multiple bioreactor units in series to achieve a continuous production process. In this configuration, each bioreactor unit can perform different biological transformation steps, thereby enhancing the efficiency and yield of the entire system. In recent years, starvation therapy represented by GOD has been considered an effective cancer treatment strategy. GOD is a natural aerobic dehydrogenase that can oxidize glucose in tumor cells into gluconic acid and H_2_O_2_, thereby depleting the glucose content in tumor cells and achieving the goal of cutting off the necessary nutrients for the tumor and starving it (63, 64). However, the hypoxic state of the TME limits the catalytic efficiency of GOD, and its poor stability and short circulation time have also hindered clinical application (65).

Recent studies have overcome the bottleneck of GOD therapy through engineered designs. The multifunctional cascade bioreactor (HCG) developed by Xiong et al. combines self-assembled pH-responsive hydroxyethyl starch prodrug (HES), doxorubicin (DOX), copper ions (Cu²^+^), and GOD to achieve synergistic treatment responsive to the TME. In a 4T1 breast cancer mouse model, HCG combined with HBOT significantly inhibited the growth of primary tumors (approximately 80% volume suppression) and reduced the number of lung metastatic nodules (approximately 70% metastasis suppression) (45). This system enhances the generation of ROS through cascade reactions, while utilizing HBOT to improve tumor oxygenation status, promote drug penetration, and activate the immune system.

Clinical translational research further validates the potential of cascade bioreactors. The semi-permeable nanoreactor (HGS-PCVs) developed by the team led by academician Xuesi Chen at Jilin University loads glucose oxidase (GOx) and hemoglobin (Hb) through complex coacervation, triggering cascade reactions in the TME to achieve synergistic therapy of chemotherapy and ferroptosis. The research shows that HGS-PCVs combined with immune checkpoint inhibitors significantly activate CD8^+^ T cell infiltration and inhibit distal tumor growth (66). Additionally, the bismuth-based nano cascade reactor developed through collaboration between Shanghai Chest Hospital and Shanghai Polytechnic University achieves TME remodeling and immune activation in a breast cancer model through photothermal-immune synergistic effects (67).

The adjunctive role of HBOT has been confirmed in multiple studies. A randomized controlled trial conducted at the University Medical Center Utrecht in the Netherlands demonstrated that HBOT significantly alleviates fibrosis symptoms in breast cancer radiotherapy patients (reducing moderate to severe fibrosis by 33%) and improves immune cell infiltration. Mechanistic studies suggest that HBOT enhances the adhesion and killing efficiency of CAR-T cells by degrading the ECM and upregulating ICAM-1 expression, leading to a 2.1 times extension of survival in a mouse model (68).

### 4.4 Clinical research on HBOT combined with upconversion nanophotosensitizer enhancing photodynamic therapy for cancer treatment

Photodynamic therapy (PDT) has shown unique advantages in the field of cancer treatment due to its minimally invasive and selective cytotoxic properties (69). However, the hypoxic microenvironment and dense ECM of solid tumors significantly limit the penetration of photosensitizers and the efficiency of ROS generation, resulting in limited efficacy of single-agent PDT (70). HBOT provides a new approach to overcome this bottleneck by improving tumor oxygenation status and degrading the ECM (35).

The innovative design of upconversion nanophotosensitizers (UNPS) has driven the innovation of PDT technology. The UNPS system developed by Li et al. utilizes near-infrared light-triggered energy conversion combined with HBOT therapy, achieving a 70% tumor volume suppression in a 4T1 breast cancer model. The research found that HBOT reshapes the tumor ECM structure, increasing the penetration depth of nanophotosensitizers by 2.3 times, significantly enhancing the efficacy of PDT (71). Xu et al. (2023) further developed “oxygen burst” nanoparticles that, through a spatiotemporally controllable oxygen release strategy, improved the efficiency of photodynamic therapy by 3.2 times (72).

Clinical translational research has confirmed the clinical value of combination therapy. A phase I/II clinical trial conducted at the Sun Yat-sen University Cancer Center showed that when treating patients with advanced head and neck cancer using HBOT combined with UNPS-PDT, the objective response rate (ORR) reached 68%, and the median progression-free survival (PFS) was extended to 8.2 months, an 82% improvement over the PDT alone group (73). Research from the University of Erlangen-Nuremberg in Germany found that HBOT pretreatment could increase the 6-month overall survival rate of patients with glioblastoma to 74%, a 28% increase compared to historical controls (74).

The synergistic effects of novel nanomaterials and HBOT provide new options for personalized treatment. Hollow mesoporous silica nanoparticles (HMSNs) loaded with photosensitizer Ce6 developed at Ruijin Hospital, affiliated with Shanghai Jiao Tong University School of Medicine, combined with HBOT therapy for liver cancer, achieved a complete remission rate of 42% in a rabbit model without observed liver damage. This study revealed that HBOT upregulates HIF-1α to promote cellular uptake of nanoparticles and enhances PDT-induced immunogenic cell death (ICD), providing a new perspective on the synergistic mechanism of combination therapy (75).

## 5 Safety data, cost-effectiveness, and translational feasibility analysis of using HBOT in combination with biotechnology for cancer treatment

### 5.1 Analysis of safety data for using HBOT in combination with biotechnology for cancer treatment

Although the combination of HBOT with biotechnology shows therapeutic potential, its safety profile requires systematic evaluation. Recent clinical trials have indicated that adverse reactions associated with HBOT primarily include oxygen toxicity (with an incidence rate of approximately 3%-5%), barotrauma (such as middle ear barotrauma, with an incidence rate of 1%-3%), and transient visual blurring (approximately 2%). These side effects are typically mild and reversible but necessitate strict monitoring of treatment parameters (oxygen partial pressure ≤2.5 ATA, single treatment duration ≤90 minutes) to mitigate risks. In a phase II clinical trial for glioblastoma patients (NCT04332068), the incidence rate of grade 3 or higher treatment-related adverse events (TRAEs) in the group receiving HBOT combined with temozolomide was 15%, significantly lower than the conventional radiochemotherapy group (28%), and no specific severe toxicities related to HBOT (such as pulmonary oxygen toxicity or central nervous system damage) were observed (76). It is worth noting that HBOT may influence the safety of immunotherapy by modulating oxidative stress levels. For instance, in melanoma patients, the incidence of immune-related adverse events (irAEs) in the group receiving HBOT combined with PD-1 inhibitors was similar to the monotherapy group (28% vs. 25%), but the combination therapy significantly increased the objective response rate (ORR: 48% vs. 32%) (77).

Furthermore, the genotoxic potential of high-pressure oxygen has been observed, potentially causing DNA damage or chromosomal abnormalities, especially with prolonged exposure or high concentrations of oxygen. In an *in vitro* experiment investigating the effects of high-pressure oxygen on human osteoblast cells (HOB) and osteosarcoma cells (SAOS-2), it was found that the level of oxidative DNA damage significantly increased in the cells after high-pressure oxygen treatment. Specifically, the degree of DNA fragmentation in HOB cells increased by 11.99 times, and in SAOS-2 cells by 4.27 times; through comet assay analysis, HOB cells increased by 15.21 times, and SAOS-2 cells by 7.33 times. These results suggest that high-pressure oxygen has the potential for genotoxicity, potentially causing damage to cellular genetic material, especially under specific experimental conditions (78). Therefore, despite the promising prospects of HBOT in cancer treatment, further research is needed to investigate its safety, efficacy, and personalized treatment strategies.

### 5.2 Cost-effectiveness and translational feasibility analysis of using HBOT in combination with biotechnology for cancer treatment

The cost-effectiveness analysis of HBOT combined with bioengineering technologies requires comprehensive consideration of therapeutic efficacy and long-term healthcare burdens. A Markov model-based cost-effectiveness study demonstrated that the incremental cost-effectiveness ratio (ICER) of HBOT combined with CAR-T cell therapy for advanced solid tumors was $52,000 per quality-adjusted life year (QALY), below the US health economics threshold ($100,000/QALY), suggesting potential economic feasibility (79). Another multicenter retrospective study (n=320) revealed that HBOT-assisted nanomedicine for triple-negative breast cancer reduced the 5-year recurrence rate from 45% to 28%, with a lifetime medical cost reduction of approximately $30,000 per patient. However, the average cost of CAR-T cell therapy reaches $400,000, and the combined treatment total cost may exceed $500,000 per patient, posing significant pressure on healthcare systems. Compared with traditional chemotherapy and radiotherapy, HBOT exhibits lower toxicity and better tolerability, though further optimization remains necessary in treatment costs and accessibility (80, 81).

HBOT relies on specialized equipment such as hyperbaric oxygen chambers, with only 2–3 hyperbaric oxygen therapy centers per one million people worldwide, mainly concentrated in developed countries (82). Biotechnologies, such as nanodrug production, must adhere to Good Manufacturing Practice (GMP) standards, with facility construction and quality control costs accounting for 30%-40% of the total treatment costs (83). To enhance accessibility, some studies propose distributed treatment models (such as mobile hyperbaric oxygen chambers) and regional cell preparation centers, which can reduce equipment costs by 40%-50% (84).

In summary, the combination of HBOT and biotechnologies demonstrates good safety and potential cost-effectiveness in cancer treatment. Despite the high short-term treatment costs, it is economically feasible in health economics through reducing recurrence rates and improving long-term quality of life. However, equipment accessibility and standardization of technology remain the main barriers to clinical translation, which need to be addressed by optimizing treatment models and infrastructure layout. Future research should further validate its long-term efficacy and drive technological innovations for broader clinical applications.

## 6 Discussion and outlook

This study comprehensively analyzed the application and synergistic mechanisms of HBOT combined with biotechnologies in cancer treatment. Previous studies have shown that HBOT can effectively increase the oxygen pressure in tumor tissues, improve the hypoxic conditions in the TME, and thus enhance the effectiveness of chemotherapy and immunotherapy (35, 45). This study further revealed that the combined application of HBOT with biotechnologies (including immunocell therapy, nanodrug delivery, etc.) not only effectively addresses the hypoxia issue in cancer treatment but also significantly enhances treatment outcomes by degrading the dense tumor ECM. Additionally, the article also explored the combined application of HBOT with porous silicon nanoparticles, engineered bacteria, cascading bioreactors, and upconversion nanophotosensitizers, demonstrating their potential in chemotherapy drug delivery, immunotherapy, and photodynamic therapy.

When discussing the synergistic effect of combining HBOT with biotechnologies for cancer treatment, a sequential treatment schedule was designed (refer to **Table 1**), detailing various stages from pretreatment to long-term monitoring (16, 37, 51, 60, 73). This schedule specifies the timing, treatment methods, mechanisms of action, and expected outcomes of each stage, aiming to optimize the treatment process and fully leverage the combined advantages of HBOT in improving the TME and biotechnologies to further enhance treatment outcomes and improve patient prognosis.

**Table 1:** Sequential treatment schedule for hyperbaric oxygen therapy (HBOT) combined with biotechnologies in cancer treatment.

Sequential Treatment Stages	Time Schedule	Treatment Methods	Mechanisms of Action	Expected Effects
Pre-treatment Stage	1–2 Days Before Treatment	HBOT Pretreatment (2.5ATA,90 minutes/session)	Increase oxygen pressure in tumor tissues, improve hypoxic conditions, activate tumor cell metabolism, and enhance sensitivity to subsequent treatments.	Enhance oxygenation levels in tumor tissues and reduce hypoxia-induced drug resistance.
Combined Treatment Stage	Treatment Days 1-3	1.HBOT (2.5ATA,90 minutes) 2.(1)Nanodrug Delivery (e.g., TMZ/PSi NPS)/(2)Immunocell Therapy (e.g., CAR-T Cells)/(3) Photodynamic Therapy (PDT)	(1) Nanodrug Delivery: HBOT induces ROS generation, degrades ECM, reduces tumor stiffness, and enhances penetration of nanodrugs. (2) Immunocell Therapy: HBOT degrades ECM, enhances CAR-T cell infiltration; activates immune cells, and strengthens anti-tumor immunity. (3) PDT: HBOT improves tumor oxygenation, enhances ROS generation efficiency of photosensitizers.	(1) Nanodrug Delivery: Increased accumulation of nanodrugs in tumor tissues, enhanced drug penetration depth. (2) Immunocell Therapy: Improved infiltration rate of CAR-T cells within tumors, enhanced immune response. (3) PDT: Significantly improved efficacy of PDT, increased tumor cell damage.
Follow-up Treatment Stage	Treatment Days 4-7	HBOT Once Daily (2.5ATA, 90 minutes)	Continuously improve the TME, maintain ECM degradation, and enhance the sustained action of immune cells and drugs.	Further inhibit tumor growth and reduce the risk of recurrence.
Long-term Monitoring Stage	1–3 Months After Treatment	HBOT Once Weekly (2.5ATA,90 minutes)	Sustain the improvement of the TME in the long term to prevent tumor recurrence.	Increase patient survival rates and improve quality of life.

In recent years, with the advancement of biotechnology, more innovative biotechnologies such as CRISPR gene editing, stem cell therapy, and microbiome therapy are entering the clinical research stage. The combination of HBOT with these new technologies will provide a more diverse range of treatment options for cancer therapy. HBOT, as a tool to improve the oxygenation status of the TME, when combined with these technologies, brings new possibilities for the development of precision medicine.

The combined application of CRISPR gene editing with HBOT demonstrates a new direction for precision cancer treatment. By editing tumor-related genes (such as p53, EGFR, KRAS), CRISPR technology can effectively inhibit tumor growth and metastasis, but its efficiency in a low-oxygen microenvironment is limited. HBOT can improve the oxygenation status of tumor tissues, enhancing the efficiency and safety of gene editing. Studies have shown that HBOT-improved oxygenation levels (pO_2_>30 mmHg) can increase the gene editing efficiency of CRISPR-Cas9 by 2.1 times in a glioma model (P<0.01), indicating that combination therapy can significantly improve the success rate of genetic repair and activate immune clearance function (85). Engineered bacteria driven by CRISPR technology show breakthrough potential in targeted tumor therapy. A study published in 2023 in “Science” developed CRISPR-Cas9-engineered EcN that specifically recognizes low-oxygen signals in the TME and secretes anti-tumor cytokines (such as TNF-α and IL-12) *in situ*, significantly inhibiting tumor growth in a mouse colorectal cancer model (with a tumor inhibition rate of 78%) while reducing toxicity to normal tissues (86). Another study published in “Nature Biotechnology” utilized CRISPR-edited Salmonella to deliver nanodrugs, degrading the tumor ECM and activating the STING pathway, enhancing the permeability and immunogenicity of chemotherapy drugs in pancreatic ductal adenocarcinoma, resulting in a 2.3-fold extension of survival in the combination therapy group (87). Additionally, a study in 2024 used CRISPR technology to modify probiotics to express PD-1 antibody fragments, successfully reversing the immunosuppressive state of the TME and significantly improving the response rate of solid tumor remission in synergy with CAR-T therapy (34). These advancements highlight the potential application of CRISPR-engineered bacteria in precision cancer therapy.

The combination of stem cell therapy with HBOT brings a dual effect to cancer treatment and tissue repair. Mesenchymal stem cells (MSCs) demonstrate unique advantages in improving the TME due to their secretion of cytokines and immunomodulatory capabilities (88). HBOT, by increasing oxygen supply, improves the survival of stem cells and promotes angiogenesis, thereby enhancing drug delivery efficiency and treatment effectiveness. The integration of microbiome therapy has become a recent research focus. By restoring gut microbiota balance, microbiome therapy can regulate the immune system and enhance anti-tumor immune responses (89, 90). The addition of HBOT not only improves the infiltration of immune cells in tumors but also provides better conditions for microbiome therapy to function.

Although these technologies show tremendous potential, their clinical translation still faces challenges. Future research needs to validate their safety and efficacy through large-scale, multicenter trials, and explore personalized treatment strategies and improved evaluation systems. The evaluation of the efficacy of combination therapy requires the integration of clinical data and long-term follow-up to establish a more reliable effectiveness and safety assessment system. Future research should focus on optimizing personalized treatment strategies, exploring the molecular mechanisms of HBOT in regulating the TME and ECM, integrating novel biotechnologies, and further evaluating the long-term efficacy and safety of combination therapy to optimize treatment strategies and promote more precise and efficient cancer treatment approaches. The combined application of HBOT with biotechnologies has indicated a new direction for cancer treatment, enhancing not only the effectiveness of cancer treatment but also providing new ideas for personalized cancer treatment. However, further in-depth basic research, clinical trials, and mechanism analysis are needed to address the current challenges. With the continuous emergence of new technologies and the strengthening of interdisciplinary collaboration, the combination of HBOT with biotechnologies is expected to become a new norm in cancer treatment, bringing hope to a large number of cancer patients.

## 7 Conclusion

The combined application of Hyperbaric Oxygen Therapy (HBOT) and biotechnological advancements provides a novel solution to break through the traditional therapeutic bottlenecks in cancer treatment. HBOT significantly enhances the efficacy of nanodrug delivery, immune cell infiltration, and photodynamic therapy (PDT) by improving the hypoxic state of the tumor microenvironment (TME) and degrading the dense extracellular matrix (ECM), demonstrating remarkable synergistic effects. This interdisciplinary strategy not only addresses the long-standing challenges of hypoxia and drug penetration in cancer therapy but also opens up new avenues for the development of precision medicine. The core innovation of this article lies in revealing the deep synergistic interplay between HBOT and biotechnologies at the molecular mechanism level. For instance, it highlights how managing the dynamic balance between reactive oxygen species (ROS) and matrix metalloproteinases (MMPs) can reshape the TME and how metabolic reprogramming of immune cells can enhance anti-tumor immune responses. Future research will focus on the long-term regulatory mechanisms of HBOT on the TME, optimizing personalized treatment plans, and developing novel biomaterials. This is expected to propel this cutting-edge technology from the experimental phase into clinical applications, potentially redefining the future of cancer treatment.
